# The Microstructural Status of the Corpus Callosum Is Associated with the Degree of Motor Function and Neurological Deficit in Stroke Patients

**DOI:** 10.1371/journal.pone.0122615

**Published:** 2015-04-15

**Authors:** Yongxin Li, Ping Wu, Fanrong Liang, Wenhua Huang

**Affiliations:** 1 Institute of Clinical Anatomy, School of Basic Medical Sciences, Southern Medical University, Guangzhou, China; 2 The 3^rd^ Teaching Hospital, Chengdu University of Traditional Chinese Medicine, Chengdu, Sichuan, China; University of Electronic Science and Technology of China, CHINA

## Abstract

Human neuroimaging studies and animal models have suggested that white matter damage from ischemic stroke leads to the functional and structural reorganization of perilesional and remote brain regions. However, the quantitative relationship between the transcallosal tract integrity and clinical motor performance score after stroke remains unexplored. The current study employed a tract-based spatial statistics (TBSS) analysis on diffusion tensor imaging (DTI) to investigate the relationship between white matter diffusivity changes and the clinical scores in stroke patients. Probabilistic fiber tracking was also used to identify structural connectivity patterns in the patients. Thirteen ischemic stroke patients and fifteen healthy control subjects participated in this study. TBSS analyses showed that the corpus callosum (CC) and bilateral corticospinal tracts (CST) in the stroke patients exhibited significantly decreased fractional anisotropy and increased axial and radial diffusivity compared with those of the controls. Correlation analyses revealed that the motor and neurological deficit scores in the stroke patients were associated with the value of diffusivity indices in the CC. Compared with the healthy control group, probabilistic fiber tracking analyses revealed that significant changes in the inter-hemispheric fiber connections between the left and right motor cortex in the stroke patients were primarily located in the genu and body of the CC, left anterior thalamic radiation and inferior fronto-occipital fasciculus, bilateral CST, anterior/superior corona radiate, cingulum and superior longitudinal fasciculus, strongly suggesting that ischemic induces inter-hemispheric network disturbances and disrupts the white matter fibers connecting motor regions. In conclusion, the results of the present study show that DTI-derived measures in the CC can be used to predict the severity of motor skill and neurological deficit in stroke patients. Changes in structural connectivity pattern tracking between the left and right motor areas, particularly in the body of the CC, might reflect functional reorganization and behavioral deficit.

## Introduction

Stroke is a leading cause of long-term motor disability among adults. Damages from ischemic stroke result in the functional and structural reorganization of perilesional and remote brain regions. However, assessing the post-stroke changes in white matter microstructure and the relationship between the motor outcome and microstructural status of these changes are remains the focus of active research. Obtaining a better understanding of the structural-behavioral correlation after stroke is therefore crucial to the development of effective therapies for patients.

Task-based functional magnetic resonance imaging (MRI) showed that stroke-affected hand movements are associated with enhanced neural activity in the ipsilateral motor cortical areas in these patients [1, 2]. A popular interpretation of this activation is that areas in the unaffected hemisphere adaptively compensate for damaged regions [3, 4]. Connectivity approaches were also used to explore this phenomenon to obtain a better understanding of whether the inter-hemispheric interaction was disrupted [5, 6]. A growing body of evidences suggests that pathological intra- and inter-hemispheric interactions among key motor regions are altered in stroke patients suffering from motor deficits [5–8]. For example, Carter et al. [5] observed that patients suffering from stroke-induced motor deficits showed a significant correlation between the disruption of inter-hemispheric functional connectivity and upper extremity impairment scores. In motor physiology, there is a growing awareness that disrupted inter-hemispheric functional interactions might underlie motor behavioral deficits [7].

In addition to these functional changes, structural imaging studies have shown that damage from ischemic stroke results in the structural reorganization of ipsilesional sensorimotor regions and transcallosal and corticospinal connections [9–12]. Studies concerning motor recovery after stroke showed that ipsilesional corticospinal tracts (CST) and transcallosal tracts showed significantly decreased fractional anisotropy (FA) and increased directional diffusivity compared with those of age-matched healthy controls [9, 12, 13]. Previous studies have primarily focused on the microstructural status of CST in stroke patients. Correlation analyses revealed that motor skill is associated with the diffusivity parameters of ipsilesional CST in chronic stroke patients [11, 12, 14]. Recently, inter-hemispheric connections (e.g., transcallosal tracts) have also been implicated in recovery in stroke patients [7, 10, 15, 16]. A multimodal MRI study revealed that the degeneration of transcallosal fibers connecting higher order sensorimotor regions is a relevant factor influencing cortical reorganization and motor outcome after subcortical stroke [15]. Although the structural integrity and predictive value in the motor recovery of transcallosal tracts have been previously studied, the quantitative relationship between the transcallosal tract integrity and the clinical scores after stroke remain unexplored. The corpus callosum (CC) is the largest white matter (WM) structure in the brain, connecting the homologous cortical areas of the two cerebral hemispheres and playing a critical role in the transfer of sensory, cognitive and motor information [10, 17–19]. The results of a previous study showed that elderly participants with abnormal gait had low fractional anisotropy (FA) in the genu of the CC, and these abnormalities were associated with gait function scores [16].

Therefore, the purpose of the present study was to examine the quantitative relationship between WM diffusivity changes in the CC and the clinical scores, including motor and neurological deficit scores, in stroke patients. To this end, diffusion tensor imaging (DTI) was conducted on a cohort of chronic stroke patients. DTI is an advanced technique that measures the magnitude and direction of water molecule diffusion within each voxel in an image [20]. This technique has become an increasingly effective tool for investigating WM microstructure [21]. In previous studies, the reliability and validity of tract-based DTI analysis approaches have been evaluated in well-recovered individuals with stroke [22]. Tract-based approaches provide complimentary rather than redundant information regarding integrity. Therefore, tract-based spatial statistics (TBSS) analysis method [23] was used in the present study to examine the hypothesis that WM diffusivity changes in the CC are associated with motor and neurological deficit scores in stroke patients. Furthermore, probabilistic tractography was performed in all subjects to determine the connectivity pattern tracking between the left and right motor areas. Previous studies have shown that bilateral motor areas are connected through transcallosal motor tracts, considered as structural substrates of inter-hemispheric interactions [12, 15, 18]. This fiber tracking schemes considers and explicitly represents the uncertainty in estimates of the principle diffusion direction [24]. Using the tractography method, we aimed to identify the structural connectivity pattern in the CC of stroke patients.

## Materials and Methods

### Subjects

Thirteen first-ever stroke patients (see Table 1 for clinical details) with unimanual motor deficits due to subcortical ischemic lesions were recruited from the Department of Neurology, the First Affiliated Hospital of Chengdu University of Traditional Chinese Medicine, China. These patients fulfilled the following inclusion criteria: (1) strictly subcortical lesions and absence of other WM pathology as verified through structural MRI; (2) absence of aphasia, neglect, and dementia; (3) no additional neurological or psychiatric disorders; (4) no previous or subsequent cerebral ischemia; and (5) at least 3 weeks between stroke onset and the time of study enrollment. None of the patients had undergone any other experimental therapy at the time of enrollment. Fifteen healthy adults without any history of neurological, psychiatric, or orthopedic disease served as controls. Information on all subjects is listed in Table 1.

**Table 1:** Demographic and imaging data.

Patient No.	Age (yr)	Gender	Dominant hand	Affected hand	Site of lesion	Lesion size (cm3)	Lesion duration (days)	DNS	FMA
1	69	F	R	R	L Pons/CS	0.35	135	13	90
2	73	M	R	R	L BG	1.13	132	26	80
3	43	F	R	R	L CN	0.45	25	15	92
4	71	F	R	L	R BG	2.24	32	21	88
5	62	M	R	R	L BG/CS	0.20	36	25	82
6	73	M	R	R	L BG	0.54	26	24	84
7	81	M	R	R	L BG/CS	1.35	22	23	83
8	49	M	R	R	L BG	0.38	56	24	85
9	67	M	R	R	L BG	1.18	33	26	80
10	78	M	R	R	L BG	3.12	21	19	84
11	54	F	R	R	L BG	1.19	45	26	80
12	54	F	R	R	L BG	0.28	23	24	86
13	63	M	R	R	L TH	0.29	21	25	82

Abbreviations: BG, basal ganglia; CN, caudate nucleus; CS, centrum semiovale; F, female; FMA, Fugl-Meyer Motor Assessment scale; L, left; M, male; NDS, Neurological Deficit Scores; R, right; TH, thalamus.

The study protocol was approved through the Ethics Committee of Chengdu University of Traditional Chinese Medicine (NO.2011KL-002), according to the principles of the Declaration of Helsinki. Every participant was informed of the purpose and procedure of this study. Informed consent was obtained from each participant prior to the study.

### Clinical Assessments

The following clinical scores were assessed on the day of examination to quantify the motor skill and the severity of neurological functional deficits in the stroke patients: Fugl-Meyer Motor Assessment (FMA) and Neurological Deficit Scores (NDS). The FMA is a well-designed, feasible and efficient clinical examination method that has been widely tested in stroke populations. FMA is a disease-specific objective impairment index [25]. The scores range from 0 to a maximum of 100 points. The higher scores denote milder impairments in motor function. NDS is an observational test to measure the severity of neurological functional deficit and assess the severity of stroke [26]. The NDS comprises 8 items, and the resulting scores range from 0 to 45, with lower values reflecting less severity of neurological functional deficit (mild: 0–15 points; moderate: 16–30 points; and severe: 31–45 points).

### Image Acquisition

Imaging data were collected using an 8-channel head coil on a 3T Siemens scanner (MAGNETOM Trio Tim, Siemens, Germany) at the West China Hospital MRI Center, Chengdu, China. The DTI protocol involved a spin echo planar image sequence with the following parameters: TR/TE = 6800/93 ms, FOV = 240×240 mm^2^, 50 axial slices, slice thickness = 3 mm, and in plane resolution = 1.875×1.875 mm^2^. Diffusion weighing was isotropically distributed along 30 directions (b = 1000 s/mm^2^). The diffusion-weighted images were acquired in blocks of 2 images with no diffusion weighting (B_0_). The B_0_ images served as anatomical references for motion correction. Foam cushions were used to reduce head translation movement and rotation. All acquisitions were visually inspected for imaging artifacts, and none of the participants were excluded on the basis of these artifacts.

### Imaging Processing and Statistical Analysis

#### TBSS analysis

For the TBSS analysis of the DTI data, images from one patient with a right-sided stroke (Patient no. 4) were oriented around the midsagittal plane prior to data analysis, thereby lateralizing the lesions to the left hemisphere in all patients [2, 11]. The DTI data from control subject matched to this patient were also midsagittally oriented. The DTI data were analyzed using the FMRIB Software Library (University of Oxford, FSL v5.0.1, www.fmrib.ox.ac.uk/fsl). Standard processing steps were used, as previously described [27]. First, eddy currents and head motion correction were performed using affine registration to the imaging without diffusion weighting [28]. The data were subsequently skull-stripped using the FMRIB Brain Extraction Tool (BET v2.1) [29]. Subsequently, the FMRIB Diffusion Toolbox (FDT v3.0) was used to fit the diffusion tensor and calculate the eigenvector and eigenvalue (λ_1_, λ_2_ andλ_3_) at each voxel [30]. The diffusion measures commonly used to characterize microstructural features of WM include FA, axial diffusivity (AD, corresponds toλ_1_) and radial diffusivity (RD, corresponds to (λ_2_ +λ_3_)/2). The FA metric, a rotational invariant index ranging from 0 (isotropic) to 1 (anisotropic), is often used as a quantitative biomarker for whiter matter integrity [31]. Higher FA values are interpreted as reflecting better WM microstructural integrity. AD represents the magnitude of water diffusion parallel to axons, and hence reflects axonal properties [32, 33]. RD reflects the magnitude of orthogonal diffusion in the direction of the primary eigenvalue, likely affected through the axolemma and myelin sheath [32, 34].

TBSS (part of FSL [23]) was used to perform TBSS analyses of FA between the patients and the controls. The TBSS procedure has been described in detail elsewhere [35]. Briefly, the FA images from all subjects were first aligned to a standard brain space using FMRIB Non-linear Imaging Registration Tool. Next, the mean FA images were created and thinned using a projection technique to create an average FA skeleton, representing the centers of the major tracts common to all subjects. A threshold of 0.2 was used to generate the mean skeleton. The aligned FA images from each subject were subsequently projected onto this skeleton. Moreover, the projection data were fed into general linear modeling cross-subject statistics. AD and RD skeletons were constructed using skeleton-projection parameters from the FA skeleton procedure, according to the tbss_non_FA procedure provided in FSL. The John Hopkins University (JHU) ICBM-DTI-81white matter atlas was used to label the regions showing significance between the groups [36].

To explore the potential local alteration of WM tracts, permutation-based nonparametric inference (n = 5000) was adopted to perform statistical analyses on FA, AD and RD. We first performed a between-group analysis to compare the patients and the controls. The statistical threshold was established as p_FWE_ < 0.05, with multiple comparison correction using threshold-free cluster enhancement [37]. To further explore the relationship between the regional brain white integrity and the clinical assessments scores, correlation analyses were examined between the diffusion indices (FA, AD and RD) and the behavioral scores (FMA and NDS). The significance threshold for the correlations was set at p < 0.01, based on the threshold-free cluster enhancement statistic image. To minimize the potential impact on the findings, age, gender, lesion size and lesion duration were used as covariates of no interest in all statistical analyses described above. The diffusion indices from the cluster located in the CC, where FA correlated with the FMA, were extracted. To visualize the correlation results, scatter plots were drawn to demonstrate the association between the diffusion indices values in these regions and the behavioral scores. A line representing the direction of the association was also drawn. To exhibit the consistency of the results with data flipped or not, same processing steps and statistical analyses were applied to the 12 patients with left-lateralized lesion and the controls.

#### Probabilistic Fiber Tracking

The probability distribution of fiber direction was calculated to estimate two directions per voxel [24]. This method was performed using a probabilistic tractography algorithm implemented in FSL (Probtrackx) and based on the Bayesian estimation of diffusion parameters (Bedpostx). Fiber tracking was initiated from all voxels within the seed mask in the diffusion space to generate 5000 streamline samples, with a step length of 0.5 mm, a curvature threshold of 0.2, and a maximum of 2000 steps. The regions-of-interest (ROIs) for tractography were located in the bilateral motor areas (including the primary motor cortex and supplementary motor areas), defined from the automated anatomical labeling (AAL) template. The ROIs were linearly transformed into the native space of each subject, where the Probtrackx analysis was ran. Fiber tracking was invoked in both directions, i.e., seeding from the left motor cortex and targeting the right motor cortex and vice versa.

After all tracts were calculated for each subject, the tracking results were thresholded to include only those voxels receiving more than 5.0 ×10^–3^ percent of the total streamlines sent out from the seed mask used to track the tract [15]. The purpose of the threshold setting was to reject low-probability voxels and reduce outlier-induced noise. For each subject, only those voxels present in both directions (left motor cortex to right motor cortex and right motor cortex to left cortex) were retained. The two pathways selected from each subject were averaged and transformed to the MNI 152 brain standard space to generate individual probabilistic maps. Statistical comparison of the individual probabilistic maps was performed using permutation-based nonparametric inference. The significance threshold for the comparisons was set at p < 0.05. The thresholded individual probabilistic maps were summed across the subjects to produce a summed probability map as a search volume mask to restrict the statistical results. The JHU white matter tractography atlas and Harvard-Oxford structural atlas were used for tract labeling. Significant differences in tractography distribution were observed between both groups.

## Results

### Behavioral Measures

**Fig 1:**
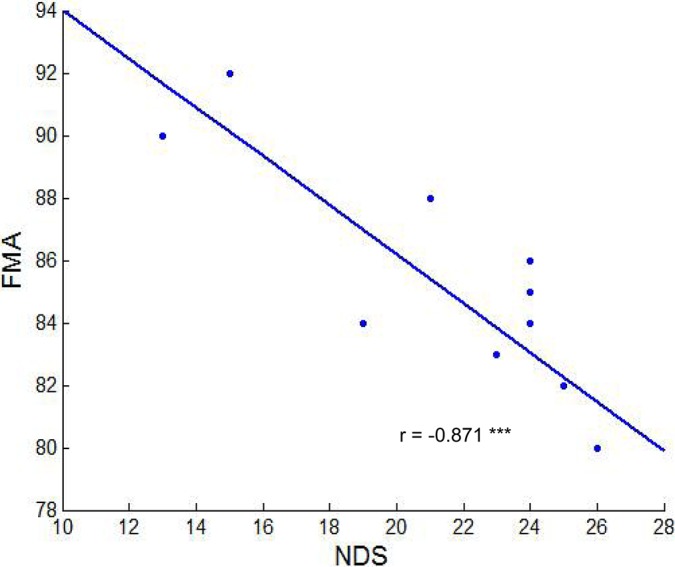
Scatter plot of correlation analysis. A significant negative correlation between Fugl-Meyer Motor Assessment (FMA) and China Neurological Deficit Scores (NDS) was observed in stroke patients (r = -0.871, p < 0.001).

Information on all subjects is listed in Table 1. Patients with ischemic stroke exhibited a range of mild to moderate neurological functional deficits. The correlation analysis revealed that the FMA scores significantly and negatively correlated with the DNS scores in these patients (Pearson’s r = -0.871, p < 0.01, Fig 1). The correlation between the behavioral performances and the lesion age/duration were also calculated. No significant correlation were found (FMA & lesion volume: Pearson’s r = -0.12; FMA & lesion duration: Pearson’s r = 0.04; NDS & lesion volume: Pearson’s r = -0.08; NDS & lesion duration: Pearson’s r = -0.23).

### TBSS Analysis of FA, Axial and Radial Diffusivity

**Fig 2:**
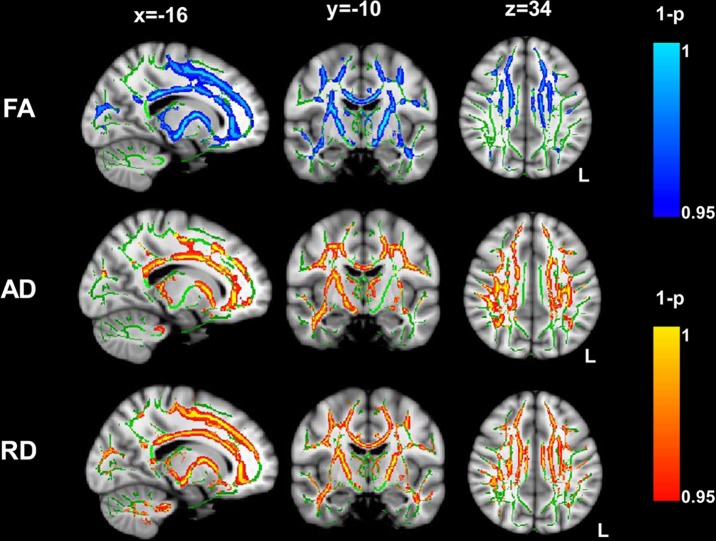
DTI-TBSS analysis showed significant areas in the stroke compared with those in the controls. White matter structures showing a significant FA decrease and an AD and RD increase in different brain regions in the stroke group (p_FWE_ < 0.05 corrected for multiple comparisons). Statistical images were overlapped onto the mean of the skeleton (green) and the MNI152 template (gray-scale) for visualization. L, left; FA, fractional anisotropy; AD, axial diffusivity; RD, radial diffusivity.

TBSS revealed a significant decrease in FA, and a significant increase in AD and RD in several brain regions in the stroke group, compared with the control group (p_FWE_ < 0.05). These regions included the CC, bilateral CST, corona radiate, limb of internal capsule and thalamic radiation (Fig 2). There was neither a significant increase in FA nor a decrease in AD and RD in the stroke patients compared with those of the controls. Similar results were found in the comparison between the only left-lateralized stroke patients and the controls (S1 Fig).

**Fig 3:**
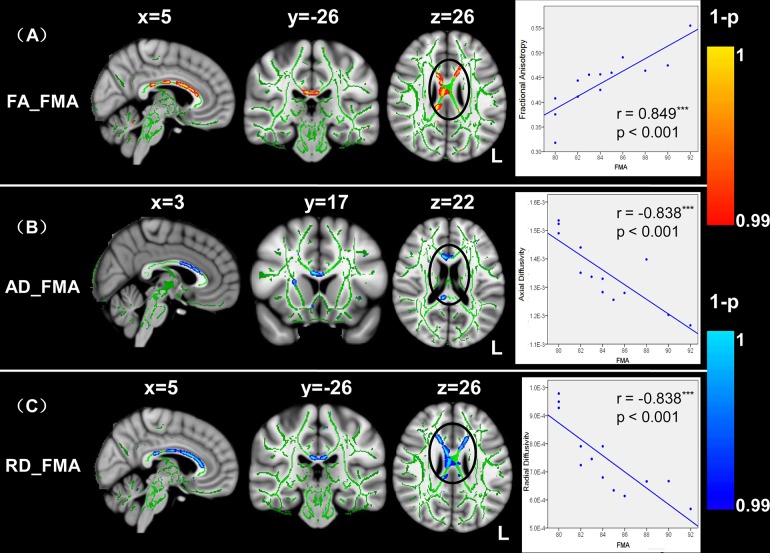
TBSS correlation analyses between FA and Fugl-Meyer Motor Assessment in patients. The FA values were positively correlated with the FMA scores (A), whereas the AD and RD values were negatively correlated with the FMA scores (B and C). DTI indices in the CC (black circle) showing consistent correlation with the FMA scores in stroke patients. The mean DTI indices from the cluster located in the CC, and the indices correlated with the FMA were extracted. Spearman correlation analyses were used and scatter plots were drawn to demonstrate associations between the mean DTI-indices and the FMA scores (rightmost pictures). The line represents the direction of the association and does not indicate a line of regression. L, left.

In the patient group, FMA were positively correlated with FA in clusters comprising the splenium and body of the CC (Fig 3A). Furthermore, FMA were negatively correlated with AD values in a number of tracts located in the genu and body of the CC and right CST (Fig 3B). Similarly, negative and significant correlations between FMA and RD were observed in the left anterior thalamic radiation, splenium, genu and body of the CC (Fig 3C). These correlations suggest that the patients with higher severity of motor deficit showed smaller FA values and larger AD and RD values in these regions, particularly the body of the CC. We extracted the mean FA values from the cluster located in the CC, where FA correlated with the FMA. To visualize the correlation results, spearman correlation analyses were used and scatter plots were drawn to demonstrate the association between the mean FA values in these regions and the FMA scores (r = 0.849, p < 0.003, see right panel of Fig 3A). The line represents the direction of the association and does not indicate a line of regression. Similar processes were performed on the AD and RD indices (Fig 3B and 3C). Spearman correlation analyses demonstrated that the mean AD and RD values were significantly and negatively correlated with FMA (AD: r = -0.838, p < 0.001; RD: r = -0.838, p < 0.001; see right panel of Fig 3B and 3C). The CC, particularly the body of CC, showed significant correlation between the diffusion indices and FMA in the only left-lateralized strokes patients (S2 Fig).

**Fig 4:**
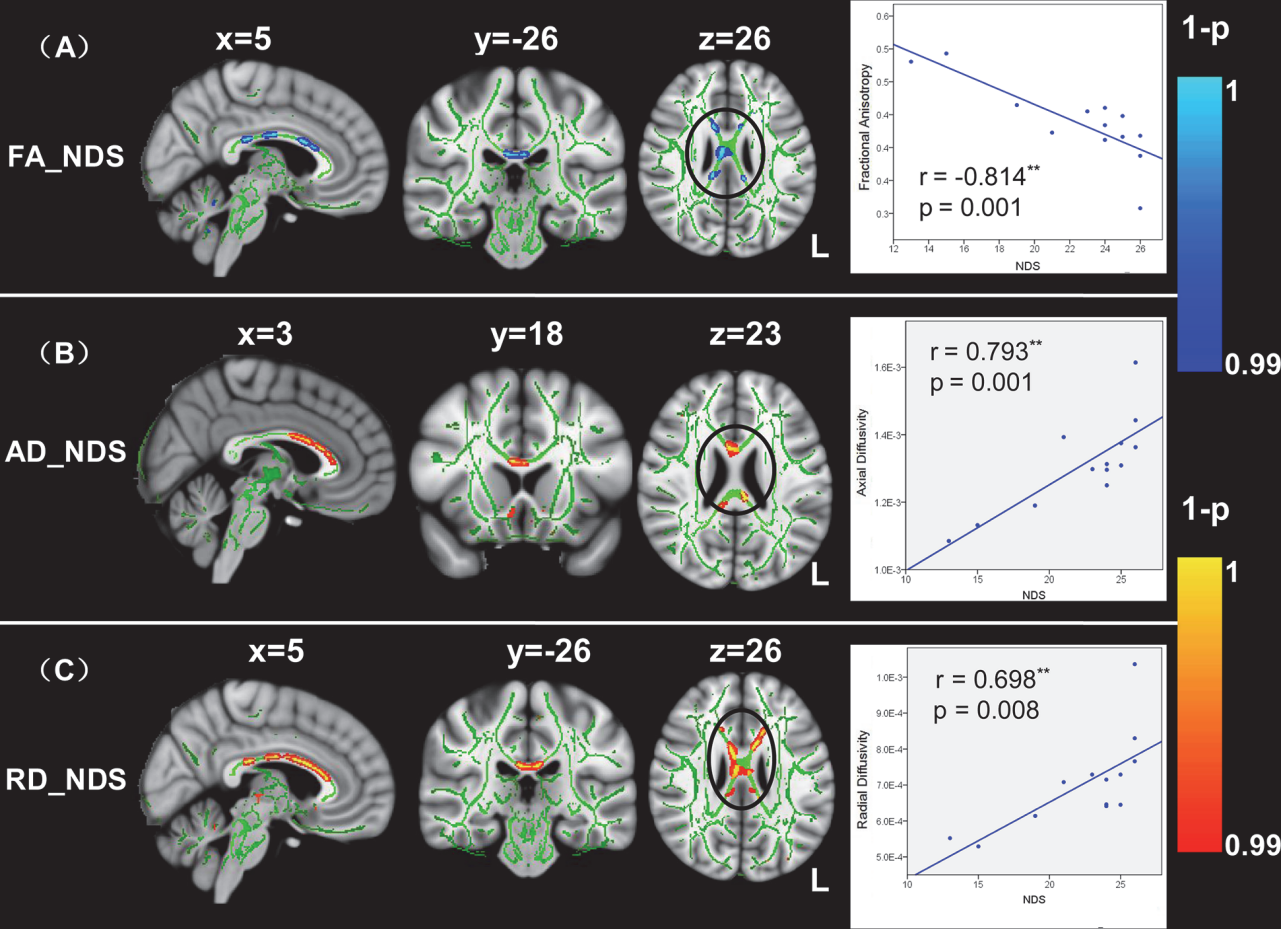
TBSS correlation analyses between FA and Neurological Deficit Scores in patients. The FA values were negatively correlated with the NDS scores (A), whereas the AD and RD values were positively correlated with the NDS scores (B and C). DTI indices in the CC (black circle) showing a consistent correlation with the NDS scores in stroke patients. The mean DTI indices from the cluster located in the CC, and the indices correlated with the NDS were extracted. Spearman correlation analyses were used, and scatter plots were drawn to demonstrate associations between the mean DTI-indices and NDS scores (rightmost pictures). The line represents the direction of the association and does not indicate a line of regression. L, left.

In the patient group, NDS were negatively correlated with FA in a number of tracts located in the splenium and body of the CC (Fig 4A). Furthermore, correlation analyses of AD and NDS showed significantly positive correlation in the anterior thalamic radiation, genu and body of the CC (Fig 4B). Similarly, positive and significant correlations between RD and NDS were observed in the bilateral CST, anterior thalamic radiation, splenium, genu and body of the CC (Fig 4C). We extracted the mean values of diffusion indices (FA, AD and RD) from the cluster located in the CC, where DTI indices correlated with the NDS. To visualize the correlation results, Spearman correlation analyses were used and scatter plots were drawn to demonstrate the association between the mean FA in these regions and the NDS scores (r = -0.814, p = 0.001; see right panel of Fig 4A), whereas mean AD and RD values were significantly and positively correlated with the NDS (AD: r = 0.793, p = 0.001; RD: r = 0.698, p = 0.008; see right panel of Fig 4B and 4C). The CC, particularly the body of CC, showed significant correlation between the diffusion indices and NDS in the only left-lateralized strokes patients (S3 Fig).

### Probabilistic Fiber Tracking

**Fig 5:**
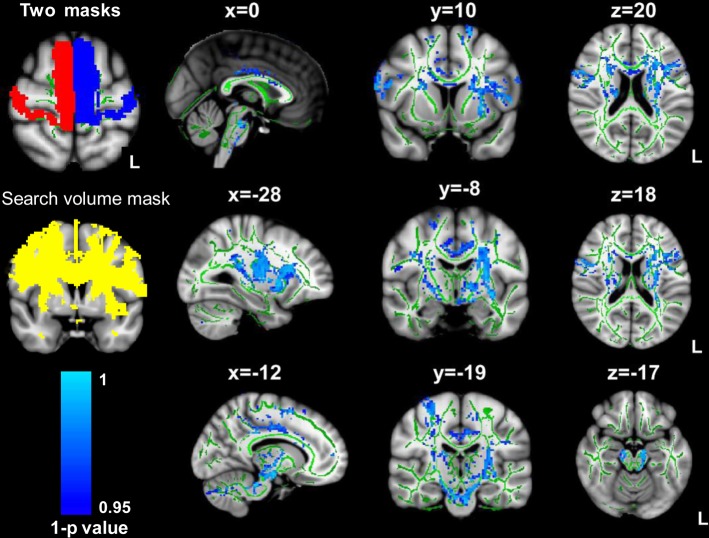
Statistical comparison of the individual probabilistic maps between groups. The statistical comparison demonstrated that the tracts connecting left and right motor regions were different between the groups. Compared with the healthy control group, a lower streamline density was detected in the patient group (read to yellow). The observed differences between the two groups were primarily located in the genu and body of the CC, left anterior thalamic radiation and inferior fronto-occipital fasciculus, bilateral CST, anterior/superior corona radiata, cingulum and superior longitudinal fasciculus (SLF). L, left.

Probabilistic fiber tracking and a multiple fiber model were used to identify the pathways connecting to the left and right motor regions in both groups. The statistical comparison results demonstrated that the tracts connecting left and right motor regions were different between groups. Compared with the healthy control group, a lower streamline density was detected in the patient group. The different projection pathways between the patient group and the controls were primarily located in the genu and body of the CC, left anterior thalamic radiation and inferior fronto-occipital fasciculus, bilateral CST, anterior/superior corona radiata, cingulum and superior longitudinal fasciculus (SLF) (see Fig 5).

## Discussion

Three principal findings were observed in the present study. First, using the TBSS method, we observed that the diffusivity pattern was altered in the CC and bilateral CST of the stroke patients with decreased FA values and increased directional diffusivities compared with those of the control group. These changes in diffusivity pattern suggest that structural changes in ipsilesional and contralesional WM occurred after stroke [11, 12]. Second, the FA values in the CC of the stroke patients were significantly correlated with the NDS and FMA scores. Similarly, correlations between the diffusion indices (such as AD and RD values) in the CC and behavior scores were detected in the stroke patients. The finding that the diffusivity-behavior correlation occurred in the CC supports the suggestion that transcallosal tracts might play an important role in measuring the severity of brain functional deficit after unilateral stroke [2, 15]. Third, probabilistic fiber tracking analyses revealed that the fiber pathways connected to the left and right motor regions in the stroke patients were significantly changed compared with those of the healthy controls. A lower streamline density was detected in the genu and body of the CC, left anterior thalamic radiation and inferior fronto-occipital fasciculus, bilateral CST, anterior/superior corona radiata, cingulum and SLF. These results suggest that changes in structural connectivity patterns might be associated with functional reorganization in stroke patients [2, 18].

### Alteration of diffusion indices

FA reflects the degree of diffusion anisotropy within a voxel, associated with WM microstructure properties, such as axonal fiber density, axonal diameter, and degree of myelination [38]. Alternations in AD are associated with axon morphological changes, whereas RD implicates the character of the myelin [32, 39]. AD and RD are the most important indices associated with FA [40]. Thus, it is better to study the changes of RD and AD to understand the mechanisms of FA change. In general, a higher FA value has been associated with improved performance, and reduced FA has been observed in neurological or psychiatric disorders [13, 16, 41–43]. Studies of stroke or other diseases have shown decreases in FA, and alternations in different patterns of AD and RD. Changes in three broad combinations of diffusion indices have been described: (1) increased RD, unchanged AD, and decreased FA; (2) increased RD, decreased AD and FA; and (3) increased RD and AD, decreased FA [44]. The results of the present study were consistent with the last combination described above. In a recent study, Saini et al. [45] showed a decrease in FA with increased AD and RD in the WM of progressive supranuclear palsy disease patients. Similar results were reported in previous studies in stroke patients [11, 12]. The results obtained from animal models of ischemia using DTI have demonstrated that changes in axial and radial diffusivity are associated with alterations in both axonal integrity and degradation of myelin [32, 33]. The observation of reduced FA with elevated AD and RD suggests a loss in the integrity of the axolemma and/or myelin sheath along the bilateral CST and CC. Ischemia might cause both axon and myelin damage in WM.

Notably, the changes in the other patterns of diffusion indices have also been observed in animal and human studies of stroke. For example, Chen et al. [13] showed the first combination of diffusion indices in the WM of ischemic stroke patients, which these authors attributed to the remodeling of the remaining axons. Sufficient remodeling in the remaining axons might occur, and thus there are no detectable barriers to AD. The second combination of diffusion indices was also detected in the studies of ischemic rat [32, 33] and subcortical stroke patients [15]. Taken together, these results showed considerable diversity in the way in which the brain regions respond to stroke disease. One explanation for the diversity of diffusivity indices might be that FA shows less sensitivity than AD and RD [40]. A longitudinal DTI study on stroke patients showed the dynamic evolution of the diffusion indices [46], prompting us to examine the diversity of the changes in diffusion indices in the future. A limitation of the present study is the sole use of adult or elderly participants. Previous studies have shown that the mean FA and AD values in the CC of newborns with acute cerebral hemisphere lesions were lower than the normal controls [47]. This pattern is different from the pattern obtained from adults in the present study. Thus, enlarging the age range to newborns might be useful to detect whether the changes in diffusion index patterns after stroke are similar between adults and newborns.

### Relations between clinical scores and diffusion index of corpus callosum

The novel findings of the present study were that diffusivity indices in the CC were significantly correlated with FMA and NDS behavioral performances in stroke patients. The CC is a major commissure connecting the cerebral hemispheres and this structure plays an important role in relaying sensory, motor, and cognitive information between homologous regions in the two cerebral hemispheres [10]. The present findings demonstrating a relationship between the microstructural status of the CC and clinical scores are consistent with the viewpoint that abnormalities of the CC are correlated with abnormalities in cognition and behavior [48, 49]. The observed DTI-behavior correlations occurred at regions along the CC, providing strong evidence for the importance of inter-hemispheric interactions for motor function and the connection integrity after stroke.

DTI-derived measures of the FA, AD and RD have been used to characterize WM damage from stroke, among other disease pathologies. FMA is the most frequently used clinical motor impairment test in stroke research [25]. In the group of ischemia stroke patients examined in the present study, we observed that diffusivity indices in the CC significantly correlated with FMA performances: patients with lower FA, higher AD and RD values were more impaired in motor function. These findings are consistent with those from previous studies in normal [48] and stroke subjects [11, 13, 15, 50]. For example, the integrity of the midbody of the CC has been associated with bimanual coordinate motor skills in normal subjects [48]. In chronic stroke, the WM integrity in the transcallosal motor tract was significant correlated with the Upper Extremity Fugl-Meyer score [13]. In addition, both cross-sectional [15] and longitudinal methods [50] were recently used to study the structural-behavioral relationship in subcortical stroke patients. The FA values in the midbody of the CC were increased in correlation with the improvement of the motor scores. In the present study, similar FA and clinical behavior correlations were detected. Furthermore, negative correlations between directional diffusivity (AD and RD) and FMA were observed, likely reflecting the changes in myelination (RD) and axon diameter (AD). Thus, we replicated and complemented these studies, providing further evidence that the structural connectivity between the inter-hemispheric motor cortex could be a marker of stroke motor impairment.

In addition to the correlation between the diffusivity indices and FMA, we also tested the relationship between the diffusivity indices and NDS to measure the severity of neurological functional deficit after stroke. Patients with higher FA and lower AD and RD values in the midbody of the CC were less impaired in neurological function. This finding is consistent with several other reports that demonstrated higher WM integrity in the CC with lower clinical severity of stroke [9, 51, 52]. Prior functional connectivity studies similarly demonstrated that inter-hemispheric motor connectivity was associated with motor functional deficit performance in stroke patients [7, 13]. Negative correlations between FMA and NDS in the present study also suggested that neurological functional deficit in stroke primarily resulted from motor function impairment. Thus, patient with serious neurological functional deficits would show a high motor impairment and a low WM integrity of the CC. Cumulatively, these findings suggested that the integrity of the midbody of the CC might play an important role in predictive motor function after stroke. Serious neurological functional deficit would destruct the effectiveness of motor information transmission between the two hemispheres.

### Building the structural connectivity pattern

Connectivity measures of networks after stroke might provide information about network reorganization. Previous functional connectivity studies consistently demonstrated system-wide network disturbances following stroke [7, 13, 53]. There is a growing evidence that stroke-induced malfunction in a brain region might spread to undamaged areas connected to lesion nodes in both hemispheres [5, 54]. Previous findings have indicated that human brain functional connectivity predictive of structural connectivity, or vice versa [55, 56]. Recently, the relationship between functional and structural connectivity has also been observed in chronic stroke patients [57]. In addition, studies of neuronal connectivity are important to establish the network underlying cognitive processes. Thus, we used a fiber-tracking method to identify whether the structural connectivity pattern was changed after stroke compared with those of the controls. In the present study, disturbed inter-hemispheric connectivity after stroke was observed at the level of microstructure. Fiber tracking analysis revealed that group structural differences were observed in the tract distribution mapping connecting the left and right motor regions. The control group showed a significantly stronger connection than the patient group in the genu and body of the CC, left anterior thalamic radiation and inferior fronto-occipital fasciculus, bilateral CST, anterior/superior corona radiate, cingulum and SLF. The observed differences in structural connectivity patterns between the two groups provide strong evidence that ischemic stroke induces brain remodeling.

Previous anatomical studies in animals and DTI studies in human revealed that the callosal motor fiber connects the primary motor cortices in the two hemispheres [16–18, 58, 59]. The callosal motor tract is located in the body of the CC. In present study, the stroke patients showed lower probability connections in the body of the CC. This finding was consistent with previous studies demonstrating that the motor CC is important for motor function. Furthermore, the anatomical location of the CC is consistent with the previous reported location of the hand callosal motor fiber [18]. As the patients recruited for the present study showed hand motor deficits, disruption of the hand callosal motor fiber might explain this stroke performance. Diffusion tractography also revealed that the middle region of the CC is connected to motor areas, whereas the posterior region is associated with parietal and temporal cortex [15, 17, 18]. Thus, damage to the fiber tracts of the body of the CC in the patients in the present study confirms an important role for the CC in motor processing and shows a different structural connection compared with that of the controls. Additionally, previous studies have shown that stroke patients suffering from motor symptoms often show damage in the CST [7, 11, 60]. Motor-related areas, such as primary motor cortex and supplementary motor area, have direct corticospinal connections to spinal cord, suggesting that the CST might play a role in motor control. Our finding that the fiber tracts in the CC and bilateral corticospinal exhibit lower probabilistic connections in stroke patients is consistent with the view that ischemic stroke might result in direct structural damage to fibers originating from bilateral motor-related areas. Other tracts, such as anterior thalamic radiation, inferior fronto-occipital fasciculus, CST, anterior/superior corona radiate, cingulum and SLF, also showed lower streamline density in stroke patients. SLF is a multiple component fiber bundle that runs in an anterior-posterior direction [61]. Previous studies have shown that this fiber bundle is functionally associated with the planning, initiation, and execution of reaching and grasping movements [62, 63]. Recent structural imaging studies in stroke patients have demonstrated that FA in the SLF was correlated with volitional and motor skill [11, 13]. Damage to all these tracts of patients in the present study confirmed the idea that the structural remodeling of functionally relevant WM tracts might be an adaptive response to stroke. Although the group differences of streamline density were found in these tracts, these results were exceeded our hypothesis. We only expect that the differences mainly located in the callosal motor fiber. Future testing with new tracking design are needed to confirm this phenomenon.

The present study also has some design characteristics that warrant further discussion. First, the stroke patients in this study included either left- or right-lateralized lesions. Patient with right-lateralized lesion was oriented around the midsagittal plane to reconcile the lesion side. When the patient with right-lateralized lesion was deleted from the statistical model, the major findings were unchanged. We can say that including the patient with right-lateralized lesion in the statistical model or not have no effect on the major findings in the present study. However, one recent functional MRI study showed that intergroup differences in brain functional connectivity were largely different between the flipped and nonflipped data [64]. Differences were also found between the left and right-lateralized patient groups in this study. Inconsistent results between our results and the study reported by Wang et al., [64] could be the result of sample size. Only one patient with right-lateralized lesion was included in the present study. More patients with right-lateralized lesion included in the study would have some effect on the results. So we think that lesion side should be considered as a factor in the future studies. Second, the lesion locations of three patients were expanded into centrum semiovale. The centrum semiovale was defined as the region comprises the central white matter core of the cerebral hemispheres. Although the part of lesions in the centrum semiovale region was small as we carefully checking, it is still a factor that might affect our fiber tracking results. Further studies are needed to take into account the location of lesion in stroke. Thirdly, as a cross-sectional study, the absence of the post-treatment measurement was a limitation. The observed DTI-behavior correlations showed that the patients with higher WM integrity would have a better behavioral performance. So we can infer that if a patient with good recovery of his motor function, the brain’s structural integrity would be improved. To further support this inference, multiple time points are needed to assess the recovery process in patients with stroke.

## Conclusion

The findings of the present study demonstrated that the diffusivity patterns in the stroke patients were changed. Specifically, the values of the DTI-derived parameters in the CC were associated with motor and neurological deficit scores in the stroke patients. The structural-behavioral relationships in the stroke patients suggested that the higher the structural integrity in the CC, the less motor and neurological functional deficit was observed. Furthermore, fiber-tracking measurements suggested that the structural abnormalities in tracts connecting the left and right motor regions confirm the hypothesis of brain remodeling after stroke. The observed correlation and fiber tracking results are consistent with the view that the integrity of the body of the CC might play an important role in the prediction of motor skill in stroke patients. These findings might enhance the current understanding of the neural processes underlying stroke and provide information about the recovery of motor function in patients. Further studies should replicate these effects using a larger sample size with functional methods to identify the specific role of the CC in stroke.

## Supporting Information

S1 Fig DTI-TBSS analysis showed significant areas in the stroke (only left-lateralized lesions) compared with those in the controls. (TIF)

S2 Fig TBSS correlation analyses between FA and Fugl-Meyer Motor Assessment in patients with only left lesions. (TIF)

S3 Fig TBSS correlation analyses between FA and Neurological Deficit Scores in patients with only left lesions. (TIF)
