# Supplementary figures and images for: The Microstructural Status of the Corpus Callosum Is Associated with the Degree of Motor Function and Neurological Deficit in Stroke Patients
Source: PLoS One. 2015 Apr 15;10(4):e0122615. doi: 10.1371/journal.pone.0122615 (PMC4398463; doi:10.1371/journal.pone.0122615)

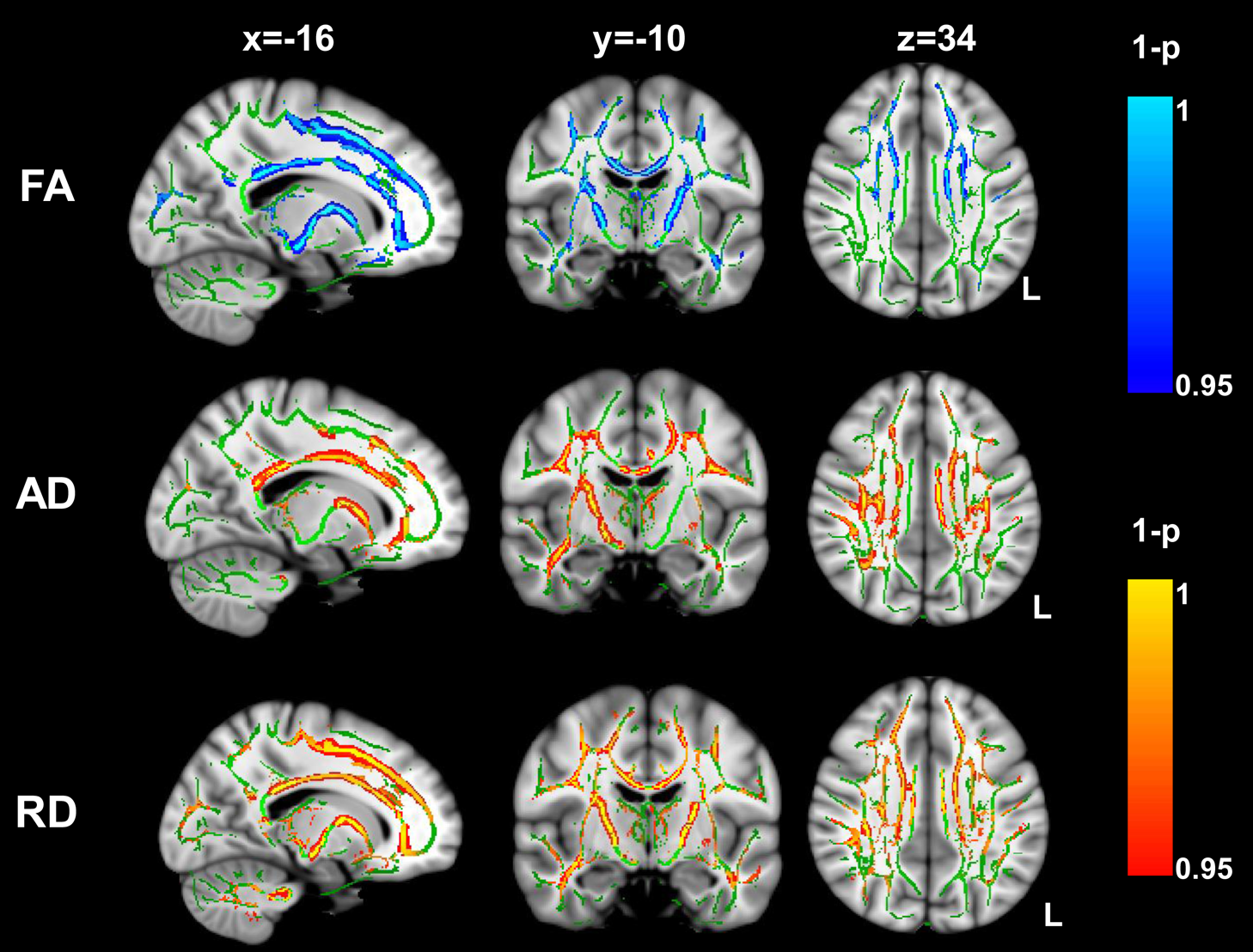

Supplement: S1 Fig — (TIF) [file pone.0122615.s001.tif]

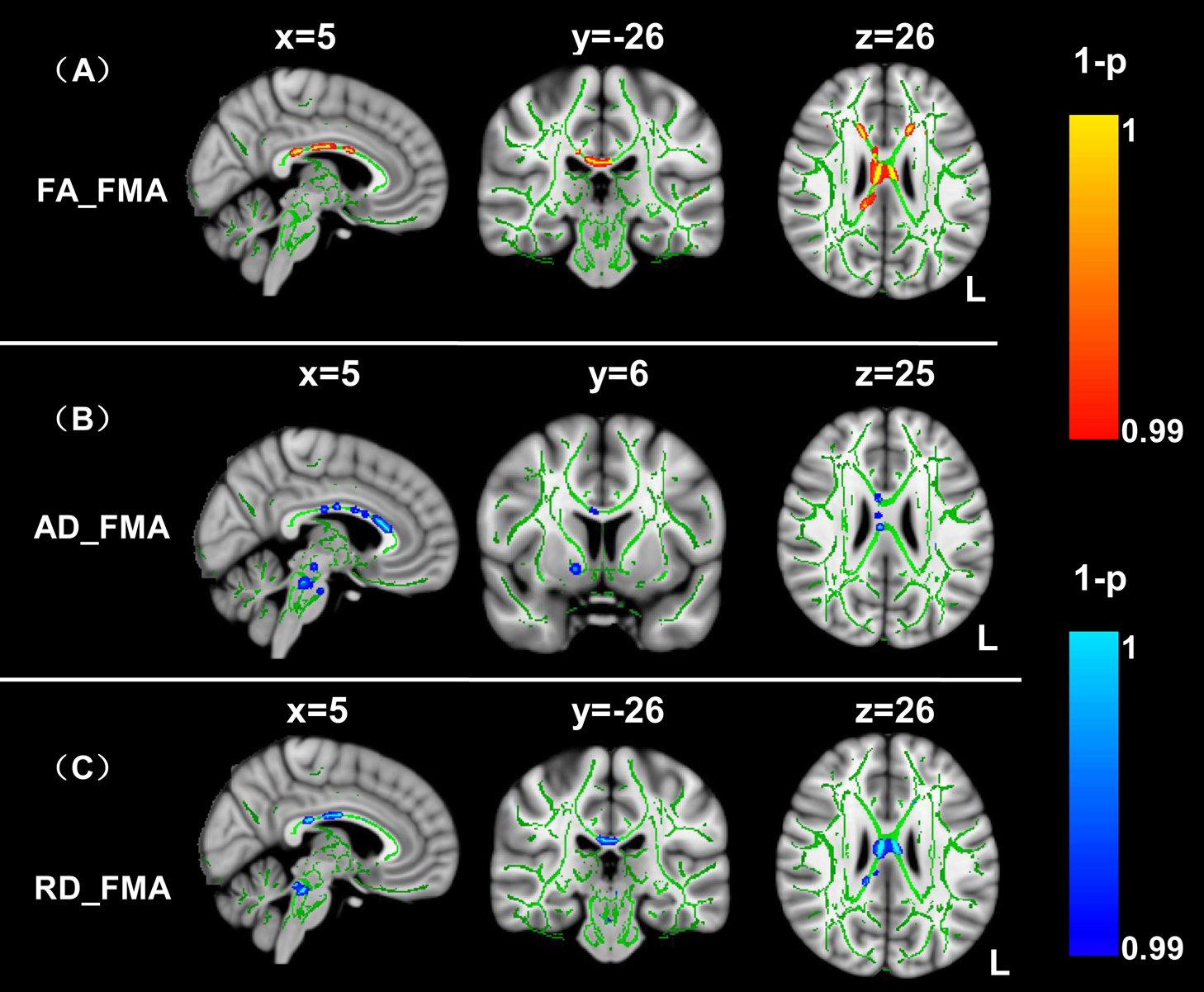

Supplement: S2 Fig — (TIF) [file pone.0122615.s002.tif]

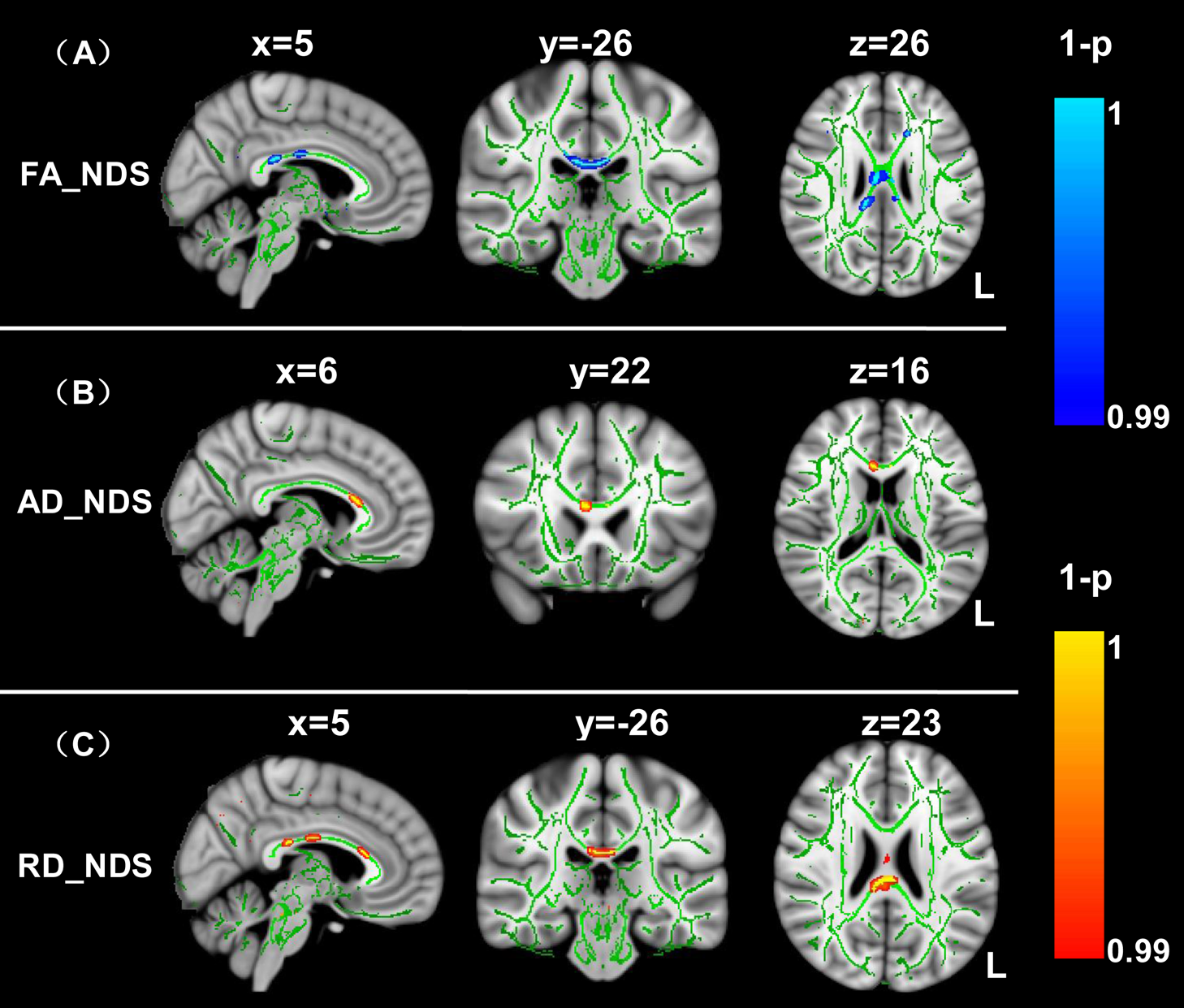

Supplement: S3 Fig — (TIF) [file pone.0122615.s003.tif]
